# Genomic analysis of *Pseudomonas* sp. GWSMS-1 isolated from Antarctica reveals its potential in Chitin hydrolysis

**DOI:** 10.1186/s12863-025-01335-0

**Published:** 2025-07-04

**Authors:** Haiyu Zeng, Zheng Wang, Jianjun Wang, Yong Yu, Wei Luo, Huirong Li, Haitao Ding

**Affiliations:** 1 School of Ecology, Hainan University, Haikou, 570228 China; 2 Polar Research Institute of China, Antarctic Great Wall Ecology National Observation and Research Station, Ministry of Natural Resources, Shanghai, 200136 China; 3 Key Laboratory for Polar Science, Polar Research Institute of China, Ministry of Natural Resources, Shanghai, 200136 China; 4 School of Oceanography, Shanghai Jiao Tong University, Shanghai, 200030 China

**Keywords:** Antarctica, Chitinase, Complete genome, Pseudomonas, Cold-adapted

## Abstract

**Objectives:**

The degradation products of chitin exhibit various biological activities, giving them significant application potential. Chitinase-producing bacteria can be isolated from diverse environments such as soil, natural waters, and rhizospheres. However, their chitinolytic activity is often limited, particularly at low temperatures.

**Data description:**

In this study, complete genome sequencing of a cold-adapted chitinolytic Pseudomonas strain, GWSMS-1, revealed a 4,606,781-bp linear chromosome with a G+C content of 59%. The genome encodes 4,599 protein-coding genes, 73 tRNA genes, and 27 rRNA genes. Functional annotation through GO, KEGG, and CAZy databases identified a substantial number of chitinase-encoding genes, which likely contribute to its high chitin-degrading capacity. The genomic insights into GWSMS-1 highlight its potential for applications in chitin degradation and offer valuable gene candidates for further research.

**Supplementary Information:**

The online version contains supplementary material available at 10.1186/s12863-025-01335-0.

## Objective

Chitin, as a natural polysaccharide substance, can be hydrolyzed into products such as N-acetylglucosamine (GlcNAc) and its dimer (GlcNAc)2 [1], which play important roles in biological systems, including structural integrity, metabolic regulation, and therapeutic applications [2]. Chitinase (EC 3.2.1.14), a glycoside hydrolase (GH) belonging to the endo-beta-1, 4-N-acetylglucosamine family, specifically hydrolyzes chitin into n-acetylglucosamine [3]. Chitinase is widely distributed in various organisms, including viruses, bacteria, fungi, plants, insects and mammals. Among these, bacterial chitinases are particularly promising due to their ease of cultivation, simple structure, and rapid propagation [4]. However, the large-scale application of chitinase is limited by its low activity, particularly at low temperatures. For example, the chitinase produced by Serratia marcescens XJ-01 exhibits significantly reduced activity in cold environments, which restricts its effectiveness in applications [5]. Similarly, the chitinase from Chitinolyticbacter meiyuanensis SYBC-H1 shows optimal activity at moderate temperatures but performs poorly in low-temperature settings [6]. Antarctica, known as a natural reservoir for c old-adapted microorganisms [7], offers a rich source of cold-adapted enzymes, which theoretically exhibit higher activity at low temperatures [8].

A chitinolytic Pseudomonas strain, GWSMS-1, was isolated from marine sediments in the Antarctic Peninsula in our previous study. When cultured on plates supplemented with colloidal chitin, GWSMS-1 produced a clear transparent zone, indicating its ability to hydrolyze colloidal chitin [9]. Native PAGE analysis of the crude enzyme secreted by strain GWSMS-1 further confirmed the presence of active chitinase, as evidenced by a prominent band on the ge; [9].

In this study, this strain was identified as a new species of Pseudomonas by comparison of 16 S rDNA similarity analysis combined with (ANI) and (dDDH) analysis. The genome characteristics of Pseudomonas were studied by comparative analysis of its source, gene number, genome size and GC content. In addition, functional annotation of the genome is used to reveal its potential for application in chitin degradation and provide important candidate genes for future research.

## Data description

The strain Pseudomonas sp. GWSMS-1 was cultivated on solid Luria-Bertani (LB) medium plate. The purified colony was subsequently transferred to liquid LB medium and cultured at 20 °C with shaking at 150 rpm until the bacterial suspension reached an optical density of 1.0 at 600 nm. The culture was centrifuged at 12,000 rpm for 5 min to collect the pellet, and genomic DNA was extracted using a Rapid Bacterial Genomic DNA Isolation Kit (Sangon Biotech - Shanghai, China) following the manufacturer’s instructions. The 16 S rDNA gene sequence of strain GWMS-1 was uploaded to EzBioCloud web server, and it showed the highest similarity (99.79%) with Pseudomonas guineae LMG 24,016 [10]. In addition, Average Nucleotide Identity (ANI) and digital DNA-DNA Hybridization (dDDH) analyses were performed using the OrthoANIu method (https://www.ezbiocloud.net/tools/ani) [11]and the Type Strain Genome Server (https://tygs.dsmz.de) [12], based on complete genome data. Strain GWSMS-1 showed ANI values of 87.61% and 84.37% with Pseudomonas guinea LMG 24,016 [10] and Pseudomonas leptonychotis CCM 8849 [13], respectively. These values fall below the 95% ANI threshold commonly used to classify strains as belonging to the same species [14]. Additionally, the dDDH values for GWSMS-1 were 57.9% with Pseudomonas guineae LMG 24,016 and 45.9% with Pseudomonas leptonychotis CCM 8849, both below the 70% threshold typically used for species delineation. The combined ANI and dDDH results strongly suggest that GWSMS-1 represents a novel species within the genus Pseudomonas. Key characteristics of Pseudomonas sp. GWSMS-1, along with the Minimum Information about a Genome Sequence (MIGS) mandatory detail, are summarized in Table S1 [15].

The genome sequence of Pseudomonas sp. GWSMS-1 was assembled using data from two sequencing platforms, 1.13 Gb from the Illumina HiSeq 2500 and 1.10 Gb from the PacBio RS II, yielding a total of 2.23 Gb of high-quality data [16]. A hybrid assembly strategy was employed, combining Illumina and PacBio reads using SPAdes v3.5.0 and Canu v1.3, respectively. Gaps in paired reads were filled using GapFiller v1.11, and PrInSeS-G v1.0.0 was used to refine the assembly and ensure accurate configuration. Genome annotation was carried out using Prokka v1.10, which integrates several predictive tools: Prodigal for coding sequences, Aragorn for tRNA, RNAmmer for rRNA, and Infernal for misc-RNA. Repeat sequences were identified and annotated using RepeatModeler and RepeatMasker (http://www.repeatmasker.org).

**Fig. 1:**
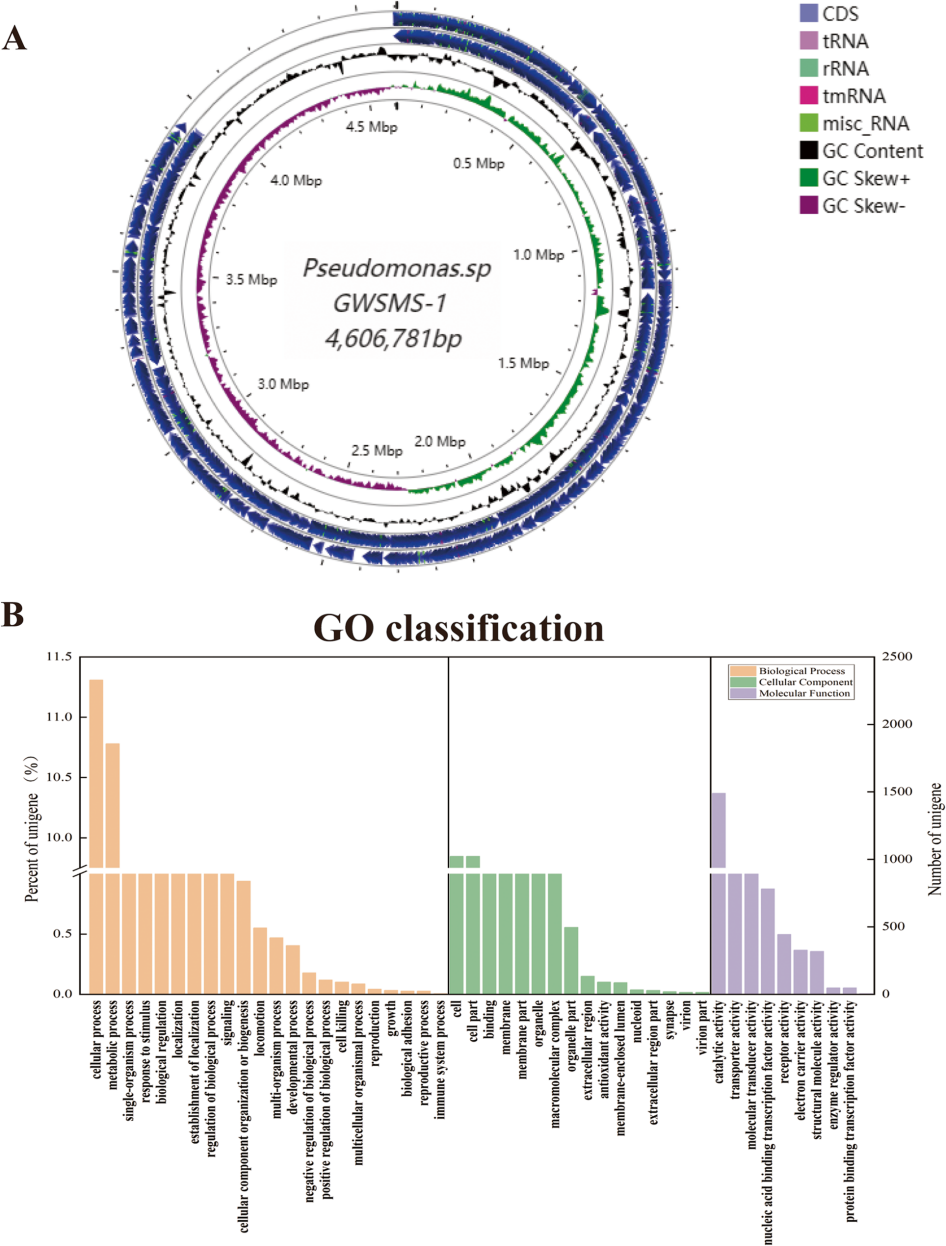
The visualization and annotation of the genome of Pseudomonas sp. GWSMS-1. Circular map of the complete genome of Pseudomonas sp. GWSMS-1 (**A**). The annotation results of GO databases were shown in (**B**)

The general genomic characteristics of Pseudomonas sp. GWSMS-1 are summarized in Table S1 [15]. The complete genome of Pseudomonas sp. GWSMS-1 consists of a 4,606,781 bp circular chromosome with a G + C content of 59%, as supported by the assembly results and the circular genome map (Fig. 1A) [17]. It encodes a total of 4,599 protein-coding genes, along with 73 tRNA genes and 27 rRNA genes.

**Fig. 2:**
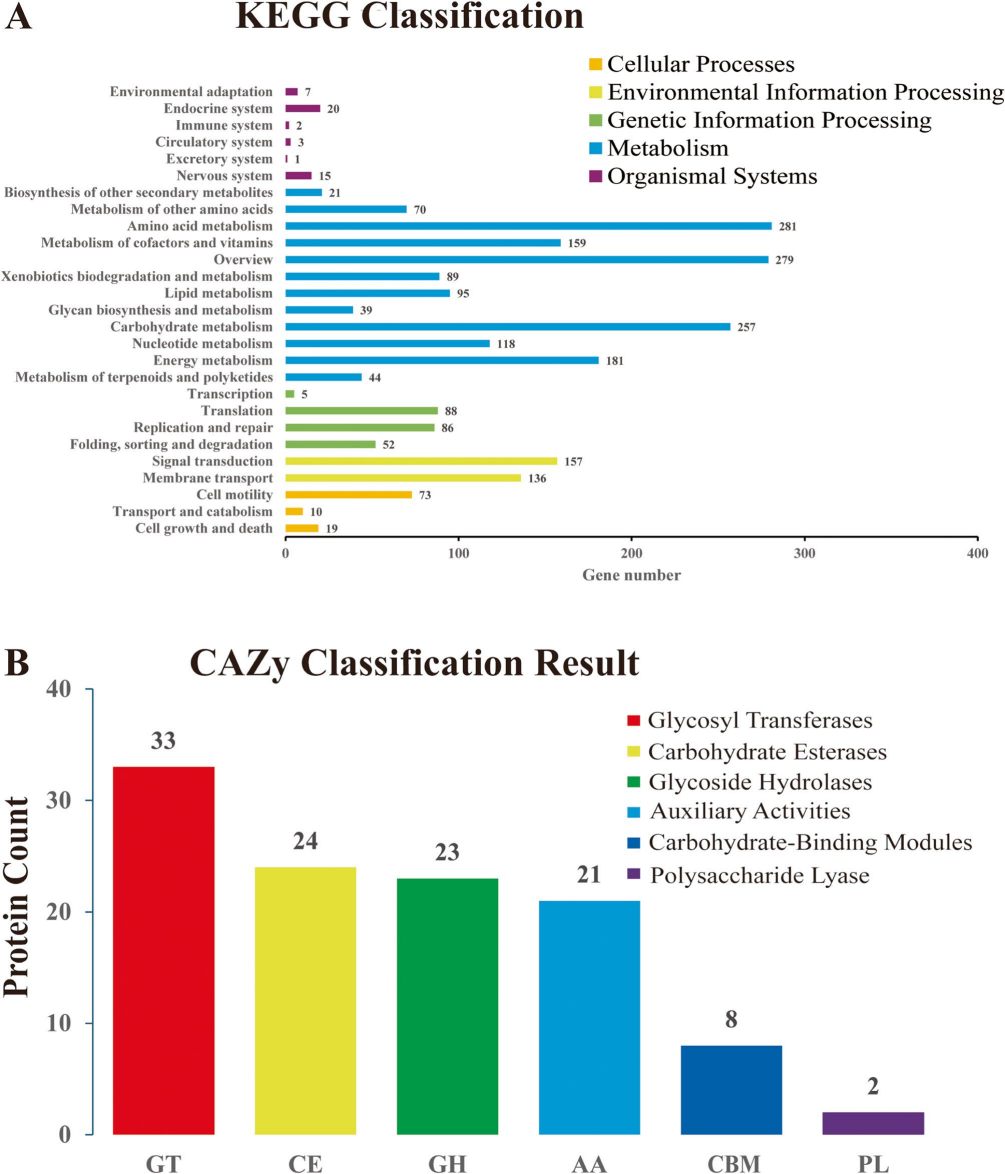
The visualization and annotation of the genome of Pseudomonas sp. GWSMS-1. The annotation results of KEEG, and CAZy databases were shown in (**A**), and (**B**), respectively

Based on Gene Ontology (GO) annotation (Fig. 1B) [17], the majority of genes in Pseudomonas sp. GWSMS-1 are associated with biological processes, with the highest gene abundance observed in cellular processes (11.30%), metabolic processes (10.77%), and single-organism processes (4.67%). Within cellular components, genes related to cell parts (9.84%) and binding (9.33%) were the most abundant. For molecular functions, catalytic activity (10.36%) and transporter activity (2.06%) dominated. Additionally, GO annotations highlighted functions such as “chitin catabolic process in ascospore wall” and “chitin deacetylase activity”, suggesting that strain GWSMS-1 has the potential to produce chitinase. Figure 2A [18] illustrates the distribution of annotated genes in KEGG pathways, showing that most genes are involved in metabolic processes, environmental information processing, and genetic information processing. Within the metabolism category, chitinase-related genes were found to play roles in amino acid metabolism (12.18%), carbohydrate metabolism (11.14%), and glycan biosynthesis and metabolism (1.69%), indicating its involvement in the degradation of complex biomolecules.

Using HMMER3 to compare the gene cluster sequences of Pseudomonas sp. GWSMS-1 with the CAZy database, it was found that the dominant enzyme classes were glycosyl transferases (GTs), carbohydrate esterases (CEs), glycoside hydrolases (GHs), and auxiliary activities (AAs) (Fig. 2B) [18]. Their respective gene sequences accounted for 29.73%, 21.62%, 20.72%, and 18.92% of the total. Strain GWSMS-1 also contains carbohydrate-related structural modules such as CBM50 and GH23. CBM50, a carbohydrate-binding module, is primarily involved in binding to polysaccharides, particularly chitin. GH23, a glycoside hydrolase family, includes enzymes commonly associated with chitinase, which are responsible for the hydrolysis and degradation of chitin.

**Fig. 3:**
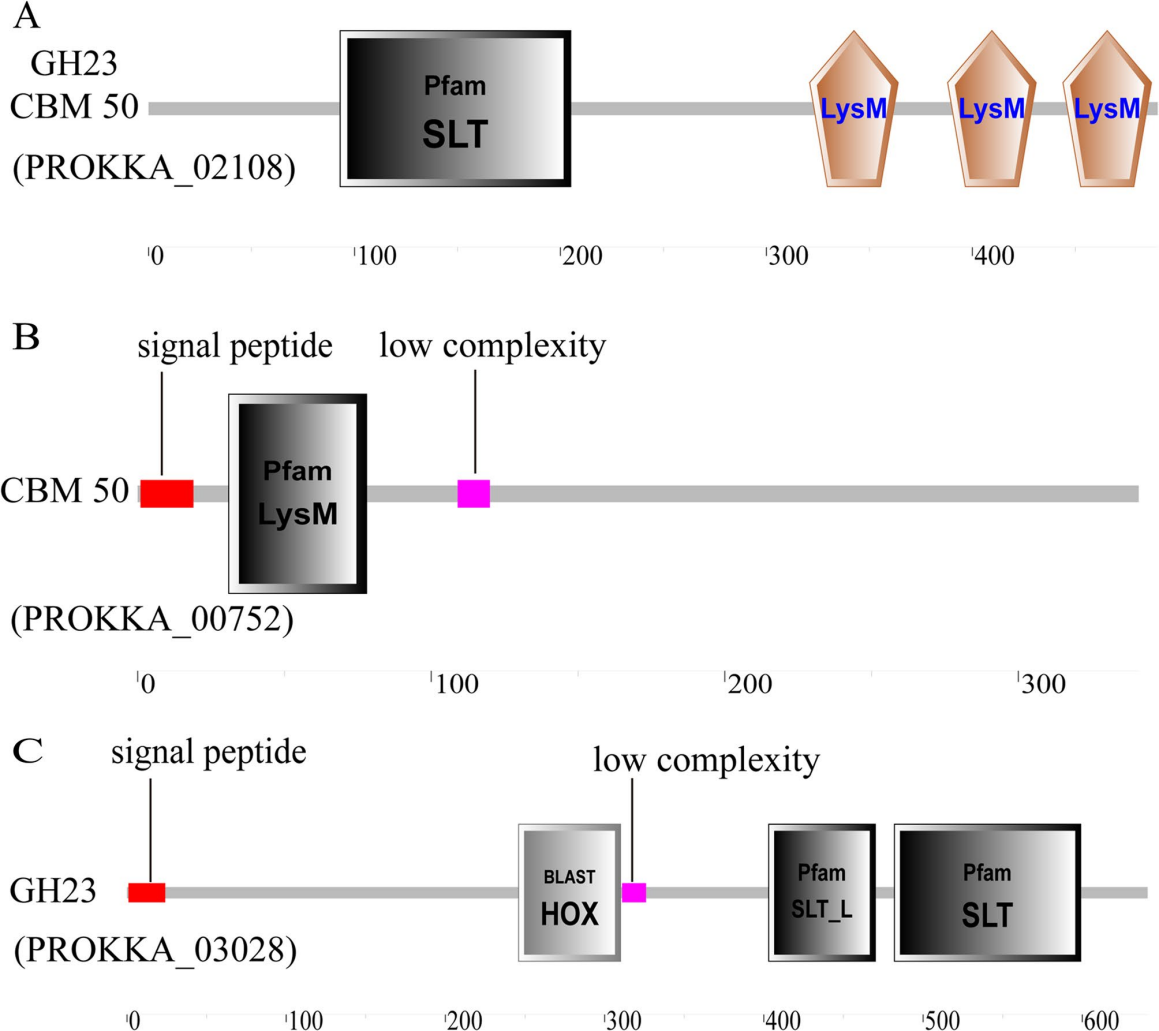
Domain architectures of chitinolytic proteins in Pseudomonas sp GWSMS-1. **A** GH23 + CM50(PROKKA_02108) (**B**) CBM50(PROKKA 00752), (**C**) GH23(PROKKA 03028)

Through comprehensive genomic analysis, we identified three key genes potentially involved in chitin degradation in Pseudomonas sp. GWSMS-1 (Table S2) [19]. The multidomain protein PROKKA_02108 (Fig. 3A) [20] contains an SLT catalytic domain (PF01464) characteristic of GH23 family enzymes, a CBM50 chitin-binding module (PF01832), and three LysM domains (PF01476), with its SLT domain showing 81.8% amino acid identity to a Pseudomonas transglycosylase (WP_411175324.1). PROKKA_03028 (Fig. 3B) [20] features an SLT domain with an N-terminal signal peptide and exhibits 99.22% sequence similarity to a Pseudomonas SLT-domain protein (WP_274088632.1), while PROKKA_00752 (Fig. 3C) [20] encodes a LysM-domain protein with 98.24% identity to a Pseudomonas LysM protein (WP_274089004.1). Structural annotation from Pfam database confirms the SLT domain belongs to glycoside hydrolase family GH23, with both CBM50 and LysM domains classified as carbohydrate-binding modules. The genomic colocalization and complementary domain architectures of these genes suggest a coordinated chitin degradation mechanism where SLT domains likely mediate hydrolytic cleavage of β−1,4-glycosidic bonds while CBM50 and LysM domains facilitate substrate recognition and binding. This functional prediction is supported by their high sequence conservation with experimentally characterized homologs and the well-documented enzymatic properties of these domain families. All sequence comparisons were performed using NCBI BLASTp with stringent parameters (e-value < 0.001), and domain annotations were verified against the Pfam database.

The genome sequence of Pseudomonas sp. GWSMS-1 provides a valuable resource for the research community, particularly for studies on cold-adapted microorganisms and their biotechnological applications. The identification of chitinase-related genes and their classification into GH families, along with domain and sequence similarity analyses, offers a foundation for further experimental validation and exploration of chitin degradation mechanisms [Table 1].

**Table 1:** Overview of data files/data sets

Label	Name of data file/data set	File types (file extension)	Data repository and identifier (DOI or accession number)
*Data file 1*	*Genome assembly of Pseudomonas sp. GWSMS-1*	*Fasta file (.fasta)*	NCBI GenBank BioProject: https://identifiers.org/ncbi/bioproject: PRJNA1153064 [16]
*Data file 2*	*General features of Pseudomonas sp. strain GWSMS-1 and MIGS mandatory information.*	*Word file (.docx)*	https://doi.org/10.6084/m9.figshare.28334012 [15]
*Data file 3*	*Circular Genome Map and Gene Ontology (GO) Annotation of Pseudomonas sp. GWSMS-1*	*Word file (.docx)*	https://doi.org/10.6084/m9.figshare.28432526 [17]
*Data file 4*	*KEGG and CAZy Database Annotations of Pseudomonas sp. GWSMS-1 Genome*	*Word file (.docx)*	https://doi.org/10.6084/m9.figshare.28675229 [18]
*Data file 5*	*Domain architectures of chitinolytic proteins in Pseudomonas sp.* *GWSMS-1*	*Word file (.docx)*	https://doi.org/10.6084/m9.figshare.28665503 [20]
*Data file 6*	*Characteristics of chitin-degrading genes in Pseudomonas sp. GWSMS-1*	*Word file (.docx)*	https://doi.org/10.6084/m9.figshare.28665590 [19]

### Limitations

The identification of GWSMS-1 as a potential new species is primarily based on 16 S rDNA similarity analysis, ANI (Average Nucleotide Identity), and dDDH (digital DNA-DNA Hybridization) values. While these genomic metrics strongly suggest novelty, a polyphasic taxonomic approach, including phenotypic and chemotaxonomic characterization, is required to formally validate GWSMS-1 as a new species. The potential for chitin hydrolysis was predicted through genomic analysis, but further experimental validation is needed to determine the chitin degradation rate, optimize degradation conditions, and evaluate the strain’s application potential in chitin degradation.As a data note, this study focuses on the description and availability of genomic data, and thus does not include experimental validation of functional predictions. Future studies should include laboratory experiments to confirm the genomic findings.

## Supplementary Information

Supplementary Material 1

Supplementary Material 2

## Data Availability

Pseudomonas sp. GWSMS-1 was deposited in the China Center for Type Culture Collection (CCTCC) with accession number CCTCC M 2019207. The complete genome assembly of Pseudomonas sp. GWSMS-1 has been deposited in the NCBI Assembly database under the accession number GCA_049847425.1. All raw sequencing data and metadata are available in the NCBI BioProject (PRJNA1153064) and BioSample (SAMN43364537) databases.

## Abbreviations

PAGE: Polyacrylamide Gel Electrophoresis
DNA: Deoxyribonucleic acid
ANI: Average Nucleotide Identity
dDDH: Digital DNA-DNA hybridization
GC: Guanine plus Cytosine content
PacBio: Pacific Biosciences
RNA: Ribonucleic Acid
GO: Gene Ontology
KEGG: Kyoto Encyclopedia of Genes and Genomes
CAZy: Carbohydrate-Active Enzymes Database
NCBI: National Center for Biotechnology Information
