# Supplementary material for: Genomic analysis of Pseudomonas sp. GWSMS-1 isolated from Antarctica reveals its potential in Chitin hydrolysis
Source: BMC Genom Data. 2025 Jul 4;26:43. doi: 10.1186/s12863-025-01335-0 (PMC12228359; doi:10.1186/s12863-025-01335-0)
Supplement: Supplementary file 1 — Supplementary Material 1 [file 12863_2025_1335_MOESM1_ESM.docx]

Table S1. General features of *Pseudomonas* sp. strain GWSMS-1 and MIGS mandatory information

| Item | Description |
| --- | --- |
| General feature  Classification | Domain Bacteria; phylum *Proteobacteria*; |
|  | Class *Gammaproteobacteria*; order *Pseudomonadales*; family *Pseudomonadaceae*; genus *Pseudomonas* |
| Gram-staining | Negative |
| Cell shape | Rod-shaped |
| Motility | Motile |
| Temperature | 20°C |
| pH | 7 |
| Salinity | 10g/L NaCl |
| Investigation type | Bacteria |
| MIGS data |  |
| project name | Complete genome sequence of *Pseudomonas* sp. GWSMS-1 |
| NCBI BioProject | PRJNA1153064 |
| NCBI BioSample | SAMN43364537 |
| Submitted to NCBI | Genbank PP550662 |
| Geographic location | Fildes Peninsula, King George Island, Antarctica |
| latitude and longitude | 62.21 S, 58.96 W |
| Collection date | 2017.1.27 |
| Environment (biome) | Marine biome (ENVO:00000447) |
| Environment (material) | Intertidal sediment (ENVO:00002179) |
| Environment (feature) | Cold environment (ENVO:01000309) |
| Isolation source | Marine sediment |
| Relationship to oxygen | Aerobic |
| Trophic level | Heterotrophic |
| Genome attributes |  |
| Sequencing platform | Illumina Hiseq and PacBio Sequel II |
| Assembly | Canu (version 1.3) and SPAdes (version 3.5.0) |
| Finishing strategy | Complete |
| Genome features |  |
| Genomic (base) | 4,606,781bp |
| Total coding gene(base) | 4,083,283bp |
| G + C content (%) | 59 |
| Protein Coding Genes | 4599 |
| tRNA | 73 |
| rRNA | 27 |
| Repeat Region Count | 269 |
| Unknown | 84 |
