# Supplementary material for: Genomic analysis of Pseudomonas sp. GWSMS-1 isolated from Antarctica reveals its potential in Chitin hydrolysis
Source: BMC Genom Data. 2025 Jul 4;26:43. doi: 10.1186/s12863-025-01335-0 (PMC12228359; doi:10.1186/s12863-025-01335-0)
Supplement: Supplementary file 2 — Supplementary Material 2 [file 12863_2025_1335_MOESM2_ESM.docx]

| Gene ID | Domain Architecture | Closest Homolog (Accession) | Identity | Predicted Function |
| --- | --- | --- | --- | --- |
| PROKKA_02108 | SLT+CBM50+3LysM | WP_411175324.1 | 81.8% | Chitin hydrolysis |
| PROKKA_03028 | SLT+Signal peptide | WP_274088632.1 | 99.22% | Secreted chitinase |
| PROKKA_00752 | LysM | WP_274089004.1 | 98.24% | Chitin recognition |

Table S2. Characteristics of chitin-degrading genes in *Pseudomonas* sp. GWSMS-1
